# Bacterial profile and antimicrobial resistance patterns of infected diabetic foot ulcers in sub-Saharan Africa: a systematic review and meta-analysis

**DOI:** 10.1038/s41598-023-41882-z

**Published:** 2023-09-05

**Authors:** Fiseha Wadilo Wada, Michael Fekadu Mekonnen, Edlawit Desta Sawiso, Sitotaw Kolato, Lideta Woldegiorgis, Gemechu Kebede Kera, Ziad El-Khatib, Akililu Alemu Ashuro, Mulatu Biru, Minyahil Tadesse Boltena

**Affiliations:** 1 Armauer Hansen Research Institute, Ministry of Health, Addis Ababa, Ethiopia; 2 Ethiopian-Evidence Based Health Care Centre: A JBI Center of Excellence, Public Health Faculty, Institute of Health, Jimma University, Jimma, Ethiopia; 3 Department of Medical Laboratory, College of Health Sciences, Wolaita Sodo University, Wolaita Sodo, Ethiopia; 4 College of Osteopathic Medicine, Kansas City University, Kansas City, USA; 5 Department of Biomedical Sciences, College of Natural and Computational Sciences, Addis Ababa University, Addis Ababa, Ethiopia; 6 Nigist Eleni Mohammed Memorial Comprehensive Specialized Hospital (NEMMCSH), Wachemo University, Hossana, Ethiopia; 7 World Health Programme, Université du Québec en Abitibi-Témiscamingue, Montreal, QC Canada; 8 Department of Global Public Health, Karolinska Institutet, Stockholm, Sweden; 9 Myungsung Medical Center, Addis Ababa, Ethiopia; 10 Jimma University Medical Center, Jimma, Ethiopia; 11 USAID Eliminate TB Project, KNCV, Addis Ababa, Ethiopia

**Keywords:** Microbiology, Medical research

## Abstract

The number of diabetic foot ulcer patients is substantially increasing, with the rapidly rising burden of diabetic mellitus in sub-Saharan Africa. The data on the regional prevalence of diabetic foot ulcer infecting bacteria and their antimicrobial resistance patterns is crucial for its proper management. This systematic review and meta-analysis determined the pooled prevalence of bacterial profiles and antimicrobial resistance patterns of infected diabetic foot ulcers in sub-Saharan Africa. A comprehensive search of the literature was performed on CINAHL, EMBASE, Google Scholar, PubMed, Scopus, and Web of Science databases. Critical appraisal was done using the Joanna Briggs Institute’s tool for prevalence studies. A pooled statistical meta-analysis was conducted using STATA Version 17.0. The *I*^*2*^ statistics and Egger’s test were used to assess the heterogeneity and publication bias. The pooled prevalence and the corresponding 95% confidence interval of bacterial profiles and their antimicrobial resistance patterns were estimated using a random effect model. Eleven studies with a total of 1174 study participants and 1701 bacteria isolates were included. The pooled prevalence of the most common bacterial isolates obtained from DFU were *S. aureus* (34.34%), *E. coli* (21.16%), and *P. aeruginosa* (20.98%). The highest pooled resistance pattern of *S. aureus* was towards Gentamicin (57.96%) and Ciprofloxacin (52.45%). *E.coli* and *K. Pneumoniae* showed more than a 50% resistance rate for the most common antibiotics tested. Both gram-positive and gram-negative bacteria were associated with diabetic foot ulcers in sub-Saharan Africa. Our findings are important for planning treatment with the appropriate antibiotics in the region. The high antimicrobial resistance prevalence rate indicates the need for context-specific effective strategies aimed at infection prevention and evidence-based alternative therapies.

## Introduction

Diabetic foot ulcer (DFU) is a severe chronic diabetic complication, which affected 15–25% of diabetic patients in their lifetime^1^. The International Diabetes Federation estimated that DFU affected 9.1 million to 26.1 million people with diabetes worldwide in 2015^2^. The global incidence of DFU has recently increased due to the increased longevity of diabetic patients and the increased prevalence of diabetes mellitus worldwide^3^. In sub-Saharan Africa (SSA), the number of DFU patients is increasing substantially, with the rapid rising of diabetes prevalence in the region^4,5^.

DFUs can progress rapidly to infection, contributing to significant morbidity and mortality in diabetic patients^6^. DFUs can be infected by different aerobes and anaerobes bacteria, Gram-positive and Gram-negative bacteria^7^. Polymicrobial DFUs infections can occur in chronic DFUs which can be colonized by different types of aerobic bacteria, such as *Staphylococcus*, *Streptococcus*, *Enterococcus*, *Pseudomonas* species, and anaerobic pathogens^7,8^. The frequency of typical microorganisms isolated from DFUs differs substantially across studies carried out in various locations throughout the world^9–12^. The bacterial distribution in DFUs can in be influenced by different factors such as geographical features, infection duration, patient’s metadata (e.g., smoking habits), and antibiotic use^7^.

According to a review conducted in 2014 by Lord Jim O'Neill and his team, it was estimated that antimicrobial resistance (AMR) has the potential to result in approximately 10 million deaths annually by the year 2050^13^. Antimicrobial resistance is a significant public health threat that has been implicated in several studies on DFUs and identified as among the key challenges to the achievement of sustainable development goals (SDG)^14–17^. A study from Kenya reported that the bacterial isolates from DFUs showed resistance to commonly used antibiotics such as ampicillin, amoxicillin, cefepime, ceftazidime, cefuroxime, clindamycin, erythromycin, piperacillin–tazobactam, tetracycline and trimethoprim–sulphamethoxazole^15^. Another study from Iran revealed that multidrug-resistant (MDR) bacteria constituted up to 48.4% of moderate-to-severe diabetic foot infections, with 37.5% of isolated *Enterococcus* species being vancomycin-resistant *Enterococcus*, 48.8% of *Staphylococcus* species. Being methicillin-resistant, 77.8% of isolated *E. coli* being ESBL and 66.7% of isolated *Pseudomonas* being MDR^17^. A recent review and meta-analysis identified ischemic ulcer, ulcer size, ulcer grade, osteomyelitis, previous antibiotic therapy and previous hospitalization as the risk factors for AMR in patients with DFU^18^.

Hence, there is a significant discrepancy in the prevalence of DFU-infecting bacteria and their AMR patterns across different regions of the world, regional data for sub-Saharan Africa is crucial for the proper management of DFUs. To date, no systematic review and meta-analysis have been conducted to investigate the prevalence and patterns of AMR in DFUs in the region. Therefore, we conducted a systematic review and meta-analysis of the literature to investigate the prevalence and patterns of AMR in DFUs in Sub-Saharan Africa.

## Methods

### Protocol registration

The International Prospective Register of Systematic Reviews (PROSPERO) has registered the study protocol for this systemic review and meta-analysis with registration code CRD42023388775^19^.

### Search strategy and selection of studies

**Figure 1:**
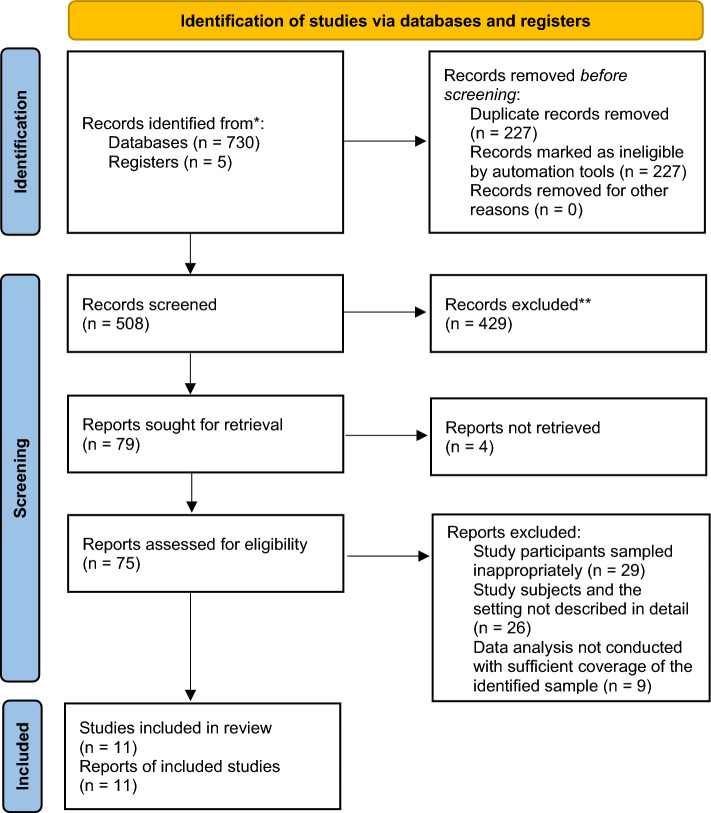
PRISMA flow diagram of included studies: Page MJ, McKenzie JE, Bossuyt PM, Boutron I, Hoffmann TC, Mulrow CD, et al. The PRISMA 2020 statement: an updated guideline for reporting systematic reviews. BMJ 2021;372:n71. https://doi.org/10.1136/bmj.n71.

This systematic review was done according to Preferred Reporting Items for Systematic Reviews and Meta-analyses (PRISMA) flow diagram (Fig. 1)^20^. The combination of MeSH/Emtree terms and free text words were used to run for each database using Boolean operators “AND” and “OR.” CINAHL, EMBASE, PubMed, Scopus, and Web of Science databases were used to retrieve the studies (supplementary material Table 1). The reference lists of all included studies were screened to obtain additional studies and authors were contacted to receive any missing articles. Original studies conducted in SSA were included without restriction on the language and year of publication^21^.

EndNote version 20.2.1. was used to remove duplicates. Two independent reviewers (FW and MTB) screened titles and abstracts, which were double-checked by a third reviewer (AAA). Potentially relevant studies were retrieved in full text and eligible studies were assessed in detail against the inclusion criteria by two reviewers (FW and MTB) and double-checked by a third reviewer (MTB). Reasons for the exclusion of studies during full text critical appraisal were recorded and reported. Discrepancies between reviewers during screening at each stage were resolved through discussion, otherwise with a third reviewer (AAA)^22^.

### Inclusion criteria

All observational studies conducted in SSA, which reported bacterial profile and/or antimicrobial resistance patterns of infected diabetic foot ulcers and published in the English language were included.

### Exclusion criteria

We excluded studies that were conducted outside SSA. Reviews, commentary, and letters to editors were also excluded. Studies that used invalid laboratory diagnostic tests and those without clear results were excluded.

### Data extraction

Data were extracted onto an Excel spreadsheet. A data extraction tool was prepared that included authors, publication year, country, healthcare setup, clinical site (ward name/clinical service area), clinical condition (disease), sample size, study design, study period, clinical sample type, bacteria identification method used, antibiotics resistance test method, standard breakpoint reference used, sex of study participants, the mean age of study participants, types and the number of bacteria isolates, and antibiotics resistance pattern. Data extraction was conducted by (FWW and MTB), and cross-checked by (AAA). In addition, there were two rounds of meetings for further data cross-checking and validation.

### Data quality and risk of bias assessment

(MTB and FW) assessed the methodological quality of eligible studies using the Joanna Briggs Institute's critical appraisal instrument for prevalence studies. The results of the critical appraisal were reported in narrative form and a table. A lower risk of bias (94%) was observed after the assessment (Table 1). Articles were reviewed using titles, abstracts, and full text screening^23^.

**Table 1:** Risk of bias assessment of 11 studies included for meta-analysis.

S. Number	Authors	Q1	Q2	Q3	Q4	Q5	Q6	Q7	Q8	Q9	Total (100)
1	Kenneth A Y et al.,2016^24^	Y	Y	Y	Y	Y	Y	Y	Y	Y	100
2	Yefou et al.,2022^25^	Y	Y	Y	Y	Y	Y	Y	Y	Y	100
3	Woldeteklie et al.,2022^26^	Y	Y	Y	Y	N	Y	Y	Y	Y	89
4	Usman et al.,2021^27^	Y	Y	N	Y	Y	Y	Y	Y	Y	89
5	Mutonga et al.,2019^15^	Y	Y	Y	Y	Y	Y	Y	N	Y	89
6	Ogba et al.,2019^28^	Y	Y	Y	Y	Y	Y	Y	Y	Y	100
7	Hamid et al.,2020^29^	Y	Y	Y	Y	Y	Y	Y	Y	Y	100
8	Ako-Nai et al.,2006^30^	Y	Y	Y	Y	Y	Y	Y	Y	Y	100
9	Berhanu et al.,2021^31^	Y	Y	Y	Y	N	Y	Y	Y	Y	89
10	Jean-Marie L et al.,2021^32^	Y	N	Y	Y	Y	Y	Y	Y	Y	89
11	Adeyemo et al.,2021^33^	Y	U	Y	Y	Y	Y	Y	Y	Y	89
	Total										94%

Y = Yes.

N = No.

U = Unknown.

### Data analysis

Data synthesis and statistical analyses were conducted using STATA version 17 software (STATA Corp., College Station, TX). The random-effect model of analysis was adopted as a method of meta-analysis because it reduced the heterogeneity of included studies. A meta-analysis of observational studies using the random-effect model of analysis was carried out. The heterogeneity was assessed using Cochrane chi-square (I^2^) statistics, while the Egger intercept was used to assess publication bias. The *P* value of < 0.05 for I^2^ statistics was used to determine the presence of heterogeneity. The findings were reported using the pooled prevalence with a 95% confidence interval (CI) and forest plot.

In line with the author's interpretation, definitions of the terms antibiotic resistance, intermediate, and susceptible were directly taken from each study. We computed the pooled prevalence of antibiotic resistance by taking absolute numbers reported by each study.

## Results

### Study selection

Systematic searching yielded 735 articles, from which 227 articles were removed due to duplication. Articles removed were (n = 508) during the title and (n = 429) abstract screening. Full-text screening involved 79, out of 68 were excluded. Only 11 articles that fulfilled the inclusion criteria were included in this systematic review and meta-analysis (Fig. 1).

### Characteristics of articles included in the meta-analysis

All the included studies were published between 2006 and 2022, out of which 4 were reported from Nigeria, 2 articles from Ethiopia, and 2 articles from Cameroon (Table 2). All studies collected swabs or biopsy samples from DFU patients to identify bacteria. Most of the included studies used a cross-sectional study design. The majority of the included studies utilized the disc diffusion method to perform antibiotic sensitivity tests, with Clinical & Laboratory Standards Institute (CLSI) guidelines serving as the standard breakpoint reference.

**Table 2:** Characteristics of articles included in the meta-analysis to assess bacterial profile and antimicrobial resistance patterns of infected diabetic foot ulcers in sub-Saharan Africa.

Authors, Year	Country	Healthcare set up	Clinical site (ward name/clinical service area)	Clinical sample type	Study design	Study period
Kenneth A Y et al.,2016^24^	Cameroon	Regional Hospital	N/M	Foot and toe wound swab culture		N/M
Yefou et al.,2022^25^	Cameroon	Central Hospital	Endoctine and Diabetlogy service	Deep wound sample culture from DFI	Cross sectional	2008–2013
Woldeteklie et al.,2022^26^	Ethiopia	Multicenter Hospitals in Addis Ababa	N/M	Leg Ulcer Swab Culture from DFU	Cross sectional	2020–2021
Usman et al.,2021^27^	Nigeria	University Teaching Hospital and General Hospital	Surgical outpatient clinic and medical ward	Ulcer Biopsies	Cross sectional	2018–2020
Mutonga et al.,2019^15^	Kenya	Tertiary Hospital			Cross sectional	2017–2018
Ogba et al.,2019^28^	Nigeria	Teritiary Health inistitution	Diabetic clinic	Foot ulcer Swab	prospective Cohort study	April—Sept 2017
Hamid et al.,2020^29^	Sudan	University Hospital	Surgery Dept	Foot ulcer Culture	crosectional Retrospective survey	2017–2019
Ako-Nai et al.,2006^30^	Nigeria	University Teaching Hospital	Medical and Surgical ward	Superficial swab and deep tissue biopsy	Prospective study	Dec 2002–March 2004
Berhanu et al.,2021^31^	Ethiopia	Public Hospital	Medical ,Orthopedic and Surgical ward.Diabetes Outpatient Clinic	Deep wound sample culture from DFI	Cross sectional	May 2018–Apr.2019
Jean-Marie L et al.,2021^32^	Congo	Hospital	Bacteriology Laboratories dept	Deep wound sample culture from DFI	N/A	2016
Adeyemo et al.,2021^33^	Nigeria	Teritiary Health center	Inpatient and out patient medical ward	Tissue biposy and Aspirates from deep seated abscesses	prospective Cross-sectional study	July 2016–April 2017

### Characteristics of the study population

Both type 1 and type 2 diabetic patients were included as the study participants. A total of 1701 bacteria were isolated from 1174 diabetic patients (Table 3). Most of the studies reported the sex of their study participants, of which 674 study participants were males and 334 were females. The mean ages of the study participants range from 54 to 62.5 years.

**Table 3:** Characteristics of study population/diabetic patients included in the meta-analysis.

Authors, Year	Clinical condition (disease)	Female participants	Male participants	Mean age (Y)	Total number of study participants	Total number of isolates
Kenneth A Y et al.,2016^24^	DM	N/R	N/R	N/R	30	30
Yefou et al.,2022^25^	DM	36	65	57.1	125	225
Woldeteklie et al.,2022^26^	T I ,TII DM	42	88	62.5	130	110
Usman et al.,2021^27^	DM patients with DFUs	81	144	54	225	172
Mutonga et al.,2019^15^	Type I AND II DM	N/R	N/R	N/R	83	80
Ogba et al.,2019^28^	DM (1 and II) patients with DFUs	31	19	55.4	50	97
Hamid et al.,2020^29^	DM (1 and II) patients with DFUs	67	183	N/R	250	335
Ako-Nai et al.,2006^30^	DM (1 and II) patients with DFUs	10	17	58	27	152
Berhanu et al.,2021^31^	Type I AND II DM	30	105	58	135	190
Jean-Marie L et al.,2021^32^	DM (1 and II) patients with DFUs	N/R	N/R	N/R	29	29
Adeyemo et al.,2021^33^	DM (1 and II) patients with DFUs	37	53	54.7	90	218
	Total	334	674		1174	1701

### Meta-analysis for the prevalence of bacteria isolates from DFU, sub-Saharan Africa

**Figure 2:**
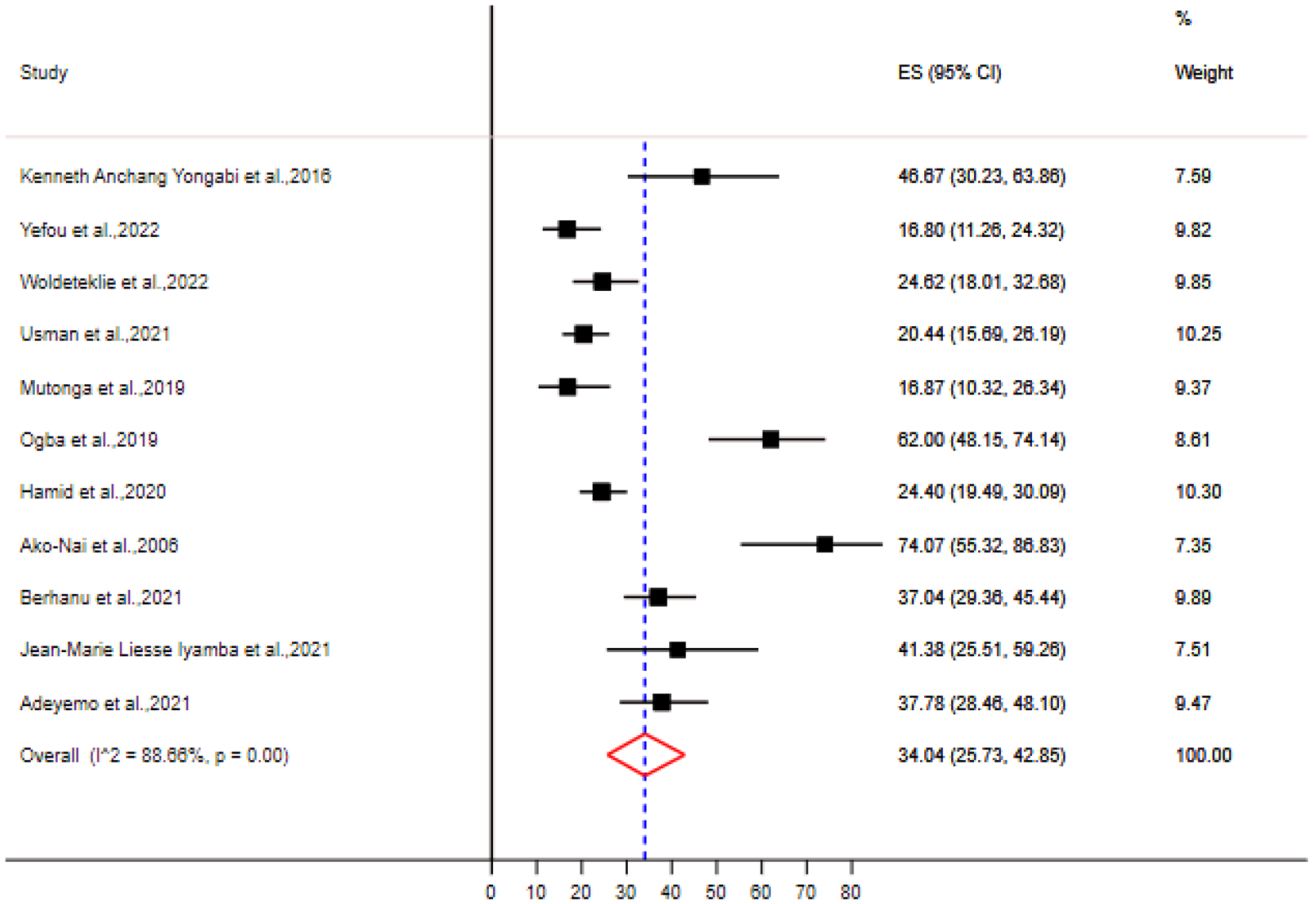
Forest plot showing the pooled prevalence of *Staphylococcus aureus* isolates from DFU samples in sub-Saharan Africa.

**Figure 3:**
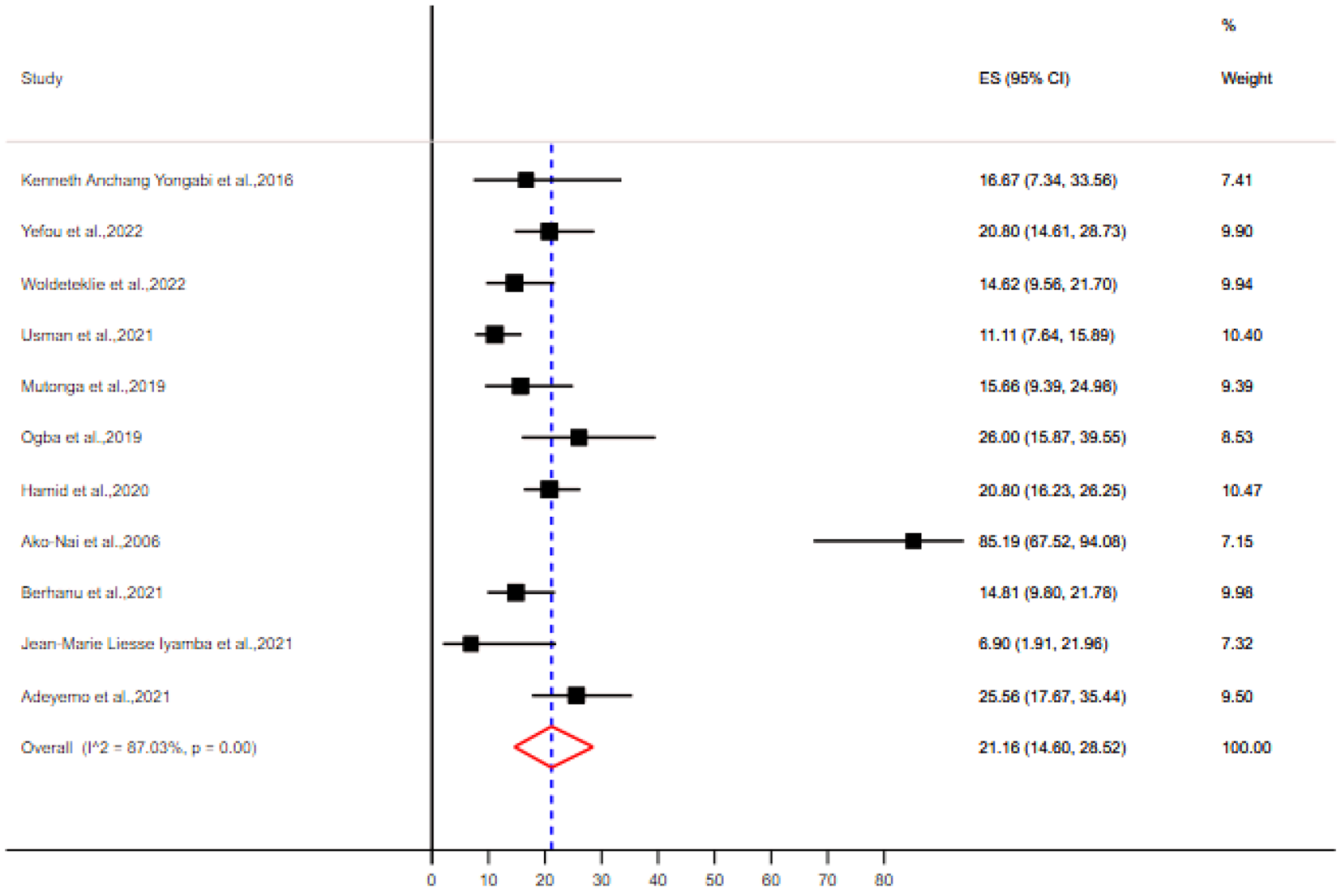
Forest plot showing the pooled prevalence of *E. coli* isolates from DFU samples in sub-Saharan Africa.

**Figure 4:**
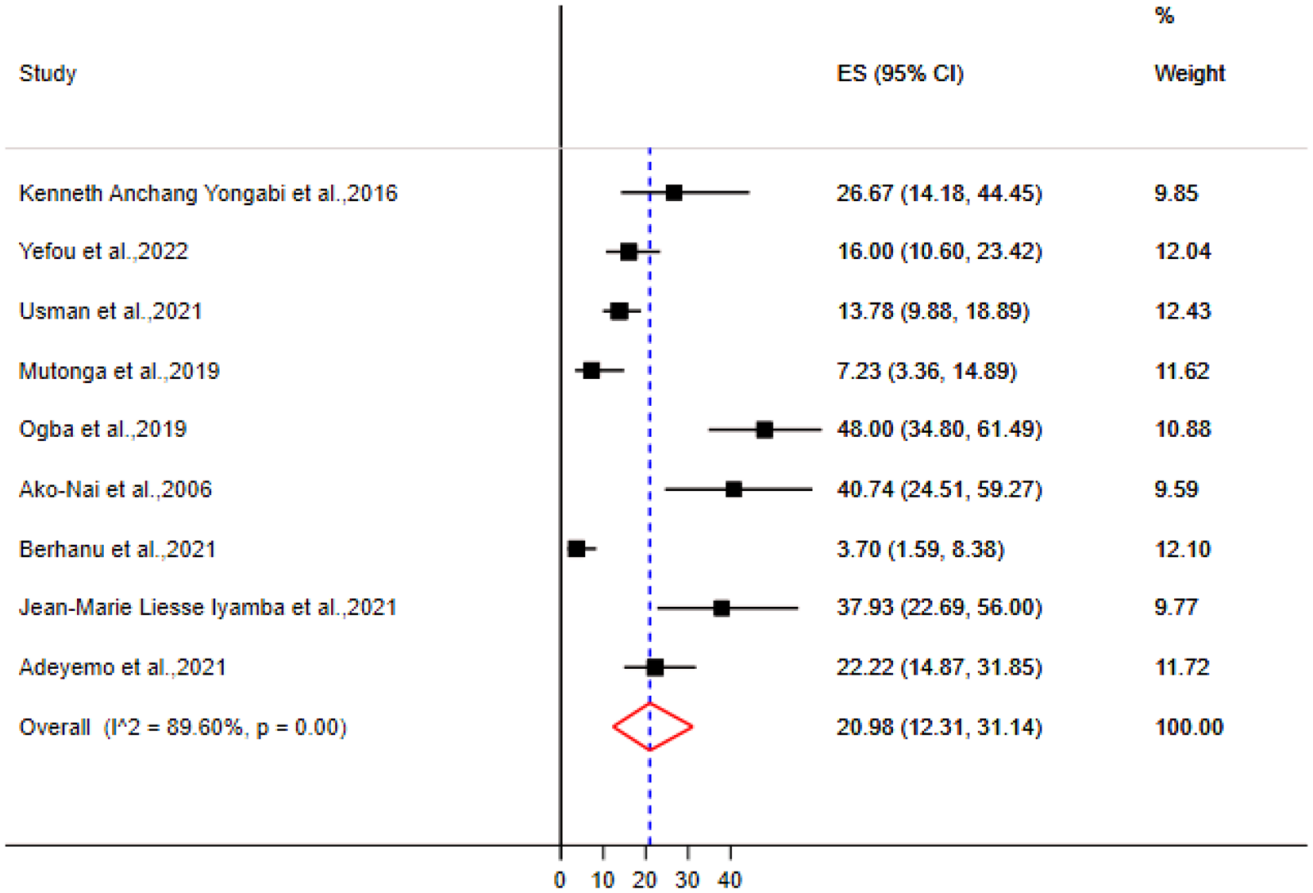
Forest plot showing the pooled prevalence of *Pseudomonas aeruginosa* isolates from DFU samples in sub-Saharan Africa.

A total of 36 bacteria species were associated with DFU in sub-Saharan Africa (Supplementary Table 1). The most prevalent gram positive bacteria was *S. aureus* (Table 4), with a pooled prevalence of 34.34% [95% CI (25.73–42.85)] (Fig. 2). The most prevalent gram negative bacteria were *E. coli* and *P. aeruginosa* with a pooled prevalence of 21.16% [95% CI (14.60–28.52)] and 20.98% [95% CI (12.31–31.14)] respectively (Figs. 3 and 4).

**Table 4:** Meta-analysis for the prevalence of bacteria isolates from DFU, sub-Saharan Africa.

Bacteria	Number of study	Number of DFU patients	Number of isolates	Pooled estimation		Heterogeneity test	
				Pooled prevalence	CI	I2	*p*-value
*S. aureus*	11	1174	335	34.34	[25.73–42.85]	88.66	< 0.01
*E. coli*	11	1174	221	21.16	[14.60–28.52]	87.03	< 0.01
*P. aeruginosa*	9	794	136	20.98	[12.31–31.14]	89.6	< 0.01
*K. Pneumoniae*	8	1027	128	11.72	[6.50–18.13]	86.92	< 0.01
*P. mirabilis*	8	770	77	12.41	[7.00–18.99]	89.15	< 0.01
*Enterococcus* species	5	825	91	9.89	[3.77–18.35]	91.72	< 0.01
*Acinetobacter* species	5	705	59	8.29	[5.26–11.89]	59.31	0.04
*M. morganii*	4	575	48	7.6	[1.07–18.76]	93.66	< 0.01
Coagulase Negative Streptococus	4	281	31	12.92	[2.16–29.70]	90.08	< 0.01
*Citrobacter* species	4	372	37	10.98	[3.15–22.33]	87.4	< 0.01
*P. vulgaris*	4	517	29	6.41	[1.65–13.54]	83.97	< 0.01
*K. oxytoca*	3	387	35	13.5	[3.21–28.77]	90.63	< 0.01
*Enterobacter* species	3	252	22				
*Streptococcus* species	2	152	25				
*E. faecalis*	2	475	56				

*S. aureus, Staphylococcus aureus; E. coli, Escherichia coli; P. aeruginosa, Pseudomonas aeruginosa; K. Pneumoniae, Klebsiella pneumoniae; P. mirabilis, Proteus mirabilis; M. morganii, Morganella morganii; P. vulgaris, Proteus vulgaris; K. oxytoca, Klebsiella oxytoca; E. faecalis, Enterococcus faecalis.*

### The pooled effect size of antibiotic resistance patterns

Among *S. aureus* isolates, the highest pooled resistance rate was toward Gentamycin (57.96%, 95% CI [40.32–74.69]), followed by Ciprofloxacin (52.45%, 95% CI [25.42–78.85]) (Table 5). Among gram negative bacteria *E.coli* and *K. Pneumoniae* were 72.42%, 95% CI [49.54–90.82] and 62.67%, 95% CI [34.32–87.41] resistance to Amoxicillin, respectively (Table 6). These bacteria also showed a higher resistance rate for Ampicillin and Ceftriaxone. *E. coli* showed the lowest resistance rate against Meropenem, with 3.06%, 95% CI [15.22–43.38].

**Table 5:** Pooled prevalence estimate of Antimicrobial Resistance Patterns against *S. aureus* from DFU in sub-Saharan Africa.

Bacteria	Antibiotics	Number of studies	Number of isolates tested	Number of resistant isolates	Pooled estimation		Heterogeneity test	
					Pooled prevalence	CI	I^2^	p-value
*S. aureus*	Ciprofloxacin	4	96	49	52.45	[25.42–78.85]	79.08	< 0.01
	Cotrimoxazole	4	130	52	39.27	[29.16–49.82]	28.40	0.24
	Gentamycin	5	131	75	57.96	[40.32–74.69]	72.13	0.01
	Erythromycin	6	180	88	45.33	[29.93–61.15]	74.67	< 0.01

**Table 6:** Pooled prevalence estimate of Antimicrobial Resistance Patterns against Gram-negative Bacteria from DFU in sub-Saharan Africa.

Bacteria	Antibiotics	Number of studies	Number of isolates tested	Number of resistant isolates	Pooled estimation		Heterogeneity test	
					Pooled prevalence	CI	I^2^	*p*-value
*E. coli*	Amoxicillin	5	104	80	72.42	[49.54–90.82]	80.39	< 0.01
	Ampicillin	7	93	60	79.96	[39.04–100.0]	92.62	< 0.01
	Cefepime	3	64	49	75.84	[38.46–99.31]	88.43	< 0.01
	Cefotaxime	4	60	34	57.37	[43.04–71.22]	0.00	0.41
	Ceftazidine	5	85	59	74.53	[43.33–97.14]	84.29	< 0.01
	Ceftriaxone	3	51	39	78.07	[55.17–94.94]	65.08	0.06
	Cefuroxime	3	46	34	75.72	[56.78–90.97]	41.81	0.18
	Ciprofloxacin	5	116	69	58.05	[43.55–71.94]	53.69	0.07
	Cotrimoxazole	4	85	65	77.21	[66.38–86.62]	14.04	0.32
	Meropenem	3	57	3	3.06	[0.00–19.67]	72.42	0.03
	Gentamicin	5	85	42	54.85	[30.46–78.27]	72.89	0.01
*P. aeruginosa*	Ceftazidine	4	39	14	32.95	[3.09–71.84]	79.56	< 0.01
	Gentamycin	5	65	31	36.47	[11.72–65.00]	76.50	< 0.01
	Ciprofloxacin	4	50	14	24.58	[8.41–44.59]	40.50	0.17
*K. Pneumoniae*	Amoxicillin	5	88	61	62.67	[34.32–87.41]	83.23	< 0.01
	Ampicillin	5	40	36	94.29	[70.51–100.0]	60.06	0.04
	Cefepime	3	46	31	63.49	[17.48–98.63]	88.68	< 0.01
	Cefotaxime	4	43	30	76.66	[26.15–100.0]	86.94	< 0.01
	Ceftazidine	5	67	52	79.09	[45.75–99.75]	82.25	< 0.01
	Ceftriaxone	3	42	37	86.86	[47.51–100.0]	84.51	< 0.01
	Cefuroxime	3	30	25	81.20	[28.94–100.0]	84.93	< 0.01
	Ciprofloxacin	5	104	42	38.31	[25.17–52.26]	40.75	0.15
	Cotrimoxazole	4	87	62	74.69	[41.26–97.59]	89.55	< 0.01
	Meropenem	3	45	13	28.38	[15.22–43.38]	0.00	0.80
	Gentamycin	6	86	40	46.11	[28.39–64.24	51.64	0.07
*P. mirabilis*	Cefuroxime	3	25	8	31.70	[13.59–52.56]	0.00	0.71
	Gentamycin	3	33	12	25.03	[0.00–70.95]	83.84	< 0.01
	Ciprofloxacin	3	33	10	28.74	[12.11–48.33]	14.87	0.31
*Enterococcus* species	Ampicillin	1	5	2				
	Erythromycin	1	13	4				
	Ticarcillin	1	16	15				
	Vancomycin	3	32	5				

### Carbapenem resistance pattern of bacterial isolates

Carbapenem-resistant bacteria are public health threats that require urgent and aggressive action. In supplementary table 2 we reported Carbapenem resistance pattern of bacterial isolates from DFU patients in sub-Saharan Africa. Some of the concerning results were reported from Congo with *P. aeruginosa* [10 (90.9%)] and *E. coli* [2 (100.0%)] resistance rates towards Imipenem^32^. Other alarming results were reported from Sudan and Ethiopia with *K. pneumonia* 7(33.3%) and 5(27.8%) resistance rate towards Meropenem, respectively^29,31^.

## Discussion

To the best of our knowledge, this is the first comprehensive review and meta-analysis conducted in sub-Saharan Africa that assesses bacterial profile and AMR patterns of DFU cases in the region. A total of 1701 bacteria were isolated from 1174 diabetic patients with DFU; the number of isolated bacteria was found very high indicating the likelihood of poly-microbial infections. *S. aureus* was found to be the most prevalent isolate obtained from DFU, followed by *E. coli* and *P. aeruginosa* in descending order of frequency. A previous worldwide meta-analysis reported diverse bacteria from diabetic foot infections, and the organism most commonly identified was *S. aureus* with a pooled prevalence estimate of 18.0% [95% CI (13.8–22.6)]^34^. A comparable composition of bacteria was also reported from the meta-analysis of general wound infection, where *S. aureus* was the most common bacterial isolate with a pooled prevalence of 36% [95% CI (29–42)] followed by *E. coli* isolates with 13% [95% CI (10–16)], *P. aeruginosa* (9% [95% CI (6–12)]), *K. pneumonia* (9% (95% CI (6–11)]) and *P. mirabilis* (8% [95% CI (5–11)])^35^.

In this meta-analysis, the pooled resistance rate of *S. aureus* towards Gentamicin and Ciprofloxacin was identified as the highest.

Compared to a study conducted in Ethiopia on general wound infections, which reported gentamicin (13% [95% CI (8–18)]) and ciprofloxacin (12% [95% CI (8–16)]) resistance rate, our results showed considerably higher differences in resistance to those antibiotics^35^. This could indicate different levels of antibiotic use or other factors that contribute to the development of antibiotic resistance in these study populations. Among gram negative bacteria *E.coli* showed more than 50% resistance rate for all antibiotics tested except for Meropenem. *K. Pneumoniae* also showed more than a 50% resistance rate for towards most of the antibiotics tested.

In February 2017, the WHO released a list of pathogens based on the growing dangers posed by antibiotic resistance that includes the pathogens designated by the acronym ESKAPE (*Enterococcus faecium, Staphylococcus aureus, Klebsiella pneumoniae, Acinetobacter baumannii, Pseudomonas aeruginosa*, and *Enterobacter* species) which were given the highest “priority status” since they represent the great threat to humans^36^. Our review also showed that these ESKAPE pathogens have a significant contribution to antibiotic resistance DFU cases in sub-Saharan Africa. In the WHO European region as well as around the globe, these pathogens were responsible for hundreds of thousands of deaths associated with antibiotic resistance^37,38^.

Infection of DFU with *Klebsiella pneumonia*, *Acinetobacter* species or *Enterobacteriaceae* may require treatment with last-resort antibiotics, such as carbapenems^39,40^. However, in our review, those pathogens showed some level of resistance to carbapenems. Therefore, these pathogens are a great threat to diabetic patients in sub-Saharan Africa as well as they are public health threats for the general population that require urgent and aggressive action.

One limitation of this meta-analysis is that it primarily focused on aerobic bacteria isolates. It is worth noting that anaerobes often play a significant role in deep tissue infections, particularly in areas with compromised vascularization due to diabetes-related microangiopathy and subsequent low oxygen tension. Additionally, it should be mentioned that most of the included studies did not report multidrug resistance patterns. Consequently, we were unable to provide an analysis of the multidrug resistance patterns exhibited by bacteria isolates of DFU.

## Conclusion

Both gram-positive and gram-negative bacteria were associated with DFU in SSA. Clinicians should be aware of bacterial resistance patterns before prescribing empirical antibiotic regimens for DFUs cases in SSA. Our findings are important for planning treatment with the appropriate antibiotics in the region. The high AMR prevalence of *E.coli* and *K. Pneumoniae* towards most of the antibiotics tested indicates the need for context-specific effective strategies aimed at practicing good hygiene and infection control measures that can help to prevent the spread of antibiotic-resistant bacteria and evidence-based alternative treatment options.

### Implication for policy and practice

Antimicrobial resistance patterns of bacteria isolated from infected diabetic foot ulcers were higher in sub-Saharan Africa. There needs to be increased focus and investment in improving the management of diabetic foot ulcers in sub-Saharan Africa. This may include the development of new treatment protocols and the provision of better resources for healthcare providers, as well as increased education and awareness for diabetic patients themselves. Additionally, there may be a need for increased research on antimicrobial resistance patterns in the region in order to inform future policy decisions related to public health and infection control. Health systems in sub-Saharan Africa must implement real-time laboratory surveillance for the identification of pathogens to determine their antimicrobial resistance profile. Countries in sub-Saharan Africa must establish a common data-sharing platform that could inform evidence regarding the antimicrobial resistance profile of ESKAPE pathogens.

### Supplementary Information

Supplementary Tables.

## Data Availability

The datasets during and/or analyzed during the current study are available from the corresponding author on reasonable request.

## Abbreviations

AMR: Antimicrobial resistance
AHRI: The Armauer hansen research institute
DFU: Diabetic foot ulcer
JBI: The Joanna Briggs institute
LMICs: Low- and middle-income countries
MDR: Multi-drug resistance
PRISMA: Preferred reporting items for systematic reviews and meta-analyses
PROSPERO: International prospective registry of systematic reviews
SDG: Sustainable development goal
SSA: Sub-Saharan Africa
WHO: The world health organization
