# Supplementary material for: Bacterial profile and antimicrobial resistance patterns of infected diabetic foot ulcers in sub-Saharan Africa: a systematic review and meta-analysis
Source: Sci Rep. 2023 Sep 5;13:14655. doi: 10.1038/s41598-023-41882-z (PMC10480146; doi:10.1038/s41598-023-41882-z)
Supplement: Supplementary file 1 — Supplementary Tables. [file 41598_2023_41882_MOESM1_ESM.docx]

**Supplementary**

**Table 1. Meta-analysis for the prevalence of bacteria isolates from DFU, sub-Saharan Africa**

| Bacteria | Number of study | Number of DFU patients | Number of isolates | Pooled estimation | | Heterogeneity test | |
| --- | --- | --- | --- | --- | --- | --- | --- |
|  |  |  |  | Pooled prevalence | CI | I2 | p-value |
| *S. aureus* | 11 | 1174 | 335 | 34.34 | [25.73-42.85] | 88.66 | <0.01 |
| *E. coli* | 11 | 1174 | 221 | 21.16 | [14.60-28.52] | 87.03 | <0.01 |
| *P. aeruginosa* | 9 | 794 | 136 | 20.98 | [12.31-31.14] | 89.6 | <0.01 |
| *K. Pneumoniae* | 8 | 1027 | 128 | 11.72 | [6.50-18.13] | 86.92 | <0.01 |
| *P. mirabilis* | 8 | 770 | 77 | 12.41 | [7.00-18.99] | 89.15 | <0.01 |
| *Enterococcus Spp* | 5 | 825 | 91 | 9.89 | [3.77-18.35] | 91.72 | <0.01 |
| *Acinetobacter spp.* | 5 | 705 | 59 | 8.29 | [5.26-11.89] | 59.31 | 0.04 |
| *M. morganii* | 4 | 575 | 48 | 7.6 | [1.07-18.76] | 93.66 | <0.01 |
| *Coagulase Negative Streptococus Spp (CONS)* | 4 | 281 | 31 | 12.92 | [2.16-29.70] | 90.08 | <0.01 |
| *Citrobacter SPP* | 4 | 372 | 37 | 10.98 | [3.15-22.33] | 87.4 | <0.01 |
| *P. vulgaris* | 4 | 517 | 29 | 6.41 | [1.65-13.54] | 83.97 | <0.01 |
| *K. oxytoca* | 3 | 387 | 35 | 13.5 | [3.21-28.77] | 90.63 | <0.01 |
| *Enterobacter Spp* | 3 | 252 | 22 |  |  |  |  |
| *Providencia Spp.* | 3 | 350 | 12 |  |  |  |  |
| *Streptococcus Spp.* | 2 | 152 | 25 |  |  |  |  |
| *Enterococcus faecalis* | 2 | 475 | 56 |  |  |  |  |
| *serratia* | 2 | 157 | 15 |  |  |  |  |
| *Staphylococcus epidermidis* | 1 | 83 | 2 |  |  |  |  |
| *Staphylococcus intermedius* | 1 | 83 | 2 |  |  |  |  |
| *Staphylococcus Simulans* | 1 | 83 | 1 |  |  |  |  |
| *Stenotrophomonas maltophilia* | 1 | 225 | 1 |  |  |  |  |
| *Klebsiella ozenae* | 1 | 135 | 8 |  |  |  |  |
| *Alcaligenes Spp* | 1 | 27 | 1 |  |  |  |  |
| *Peptostreptococcus Spp* | 1 | 27 | 4 |  |  |  |  |
| *Clostridium perfringens* | 1 | 27 | 2 |  |  |  |  |
| *Bacteriodes fragilis Spp* | 1 | 27 | 7 |  |  |  |  |
| *Fusobacterium Spp* | 1 | 27 | 1 |  |  |  |  |
| *Hafnia alvei* | 1 | 90 | 5 |  |  |  |  |
| *Serratia fonticola* | 1 | 83 | 3 |  |  |  |  |
| *Citrobacter freundii* | 1 | 225 | 6 |  |  |  |  |
| *Viridian sterptococci spp.* | 1 | 135 | 2 |  |  |  |  |
| *Salmonella Sp* | 1 | 29 | 1 |  |  |  |  |
| *Corynebacterium Spp* | 1 | 27 | 8 |  |  |  |  |
| *Proeteus Morganii* | 1 | 27 | 8 |  |  |  |  |
| *Stenotrophomonas maltophilia* | 1 | 225 | 1 |  |  |  |  |
| *Bacillus Spp.* | 1 | 30 | 2 |  |  |  |  |

**Table 2. Carbapenem resistance pattern of bacterial isolates from DFU in sub-Saharan Africa**

| Bacteria | Authors, Primary | Pub Year | Country | Antibacterial agent | Total isolates | Resistant isolates | Resistance % |
| --- | --- | --- | --- | --- | --- | --- | --- |
| Staphylococcus aureus | Hamid et al. | 2020 | Sudan | Meropenem | 1 | 0 | 0.0 |
|  | Hamid et al. | 2020 | Sudan | Imipenem | 1 | 0 | 0.0 |
|  | Jean-Marie Liesse Iyamba et al | 2021 | Congo | Imipenem | 12 | 1 | 8.3 |
| Enterococcus faecalis | Hamid et al. | 2020 | Sudan | Meropenem | 1 | 0 | 0.0 |
| Enterobacter Spp | Adeyemo et al. | 2021 | Nigeria | carbapenem | 11 | 0 | 0.0 |
| Escherichia coli | Adeyemo et al. | 2021 | Nigeria | carbapenem | 23 | 0 | 0.0 |
|  | Mutonga et al. | 2019 | Kenya | Meropenem | 13 | 3 | 23.1 |
|  | Hamid et al. | 2020 | Sudan | Meropenem | 24 | 0 | 0.0 |
|  | Berhanu et al. | 2021 | Ethiopia | Meropenem | 20 | 0 | 0.0 |
|  | Yefou et al. | 2022 | Cameroon | Imipenem | 21 | 0 | 0.0 |
|  | Hamid et al. | 2020 | Sudan | Imipenem | 20 | 0 | 0.0 |
|  | Jean-Marie Liesse Iyamba et al | 2021 | Congo | Imipenem | 2 | 2 | 100.0 |
| Klebsiella Pneumoniae | Mutonga et al. | 2019 | Kenya | Meropenem | 6 | 1 | 16.7 |
|  | Hamid et al. | 2020 | Sudan | Meropenem | 21 | 7 | 33.3 |
|  | Berhanu et al. | 2021 | Ethiopia | Meropenem | 18 | 5 | 27.8 |
|  | Yefou et al. | 2022 | Cameroon | Imipenem | 19 | 1 | 5.3 |
|  | Hamid et al. | 2020 | Sudan | Imipenem | 12 | 0 | 0.0 |
|  | Jean-Marie Liesse Iyamba et al | 2021 | Congo | Imipenem | 2 | 2 | 100.0 |
| Proteus mirabilis | Adeyemo et al. | 2021 | Nigeria | carbapenem | 10 | 0 | 0.0 |
|  | Mutonga et al. | 2019 | Kenya | Meropenem | 9 | 0 | 0.0 |
|  | Berhanu et al. | 2021 | Ethiopia | Meropenem | 7 | 0 | 0.0 |
| Proteus Spp | Hamid et al. | 2020 | Sudan | Meropenem | 19 | 1 | 5.3 |
|  | Yefou et al. | 2022 | Cameroon | Imipenem | 15 | 1 | 6.7 |
|  | Hamid et al. | 2020 | Sudan | Imipenem | 20 | 0 | 0.0 |
| Pseudomonas aeruginosa | Adeyemo et al. | 2021 | Nigeria | carbapenem | 20 | 3 | 15.0 |
|  | Mutonga et al. | 2019 | Kenya | Meropenem | 6 | 0 | 0.0 |
|  | Berhanu et al. | 2021 | Ethiopia | Meropenem | 5 | 0 | 0.0 |
|  | Yefou et al. | 2022 | Cameroon | Imipenem | 11 | 1 | 9.1 |
|  | Jean-Marie Liesse Iyamba et al | 2021 | Congo | Imipenem | 11 | 10 | 90.9 |
| Morganella morganii | Adeyemo et al. | 2021 | Nigeria | carbapenem | 7 | 1 | 14.3 |
|  | Berhanu et al. | 2021 | Ethiopia | Meropenem | 2 | 0 | 0.0 |
|  | Yefou et al. | 2022 | Cameroon | Imipenem | 19 | 3 | 15.8 |
| Coagulase Negative Streptococus Spp (CONS) | Jean-Marie Liesse Iyamba et al | 2021 | Congo | Imipenem | 1 | 0 | 0.0 |
| Salmonella Sp | Jean-Marie Liesse Iyamba et al | 2021 | Congo | Imipenem | 1 | 1 | 100.0 |
| Klebsiella Oxytoca | Berhanu et al. | 2021 | Ethiopia | Meropenem | 15 | 1 | 6.7 |
| Klebsiella Ozenae | Berhanu et al. | 2021 | Ethiopia | Meropenem | 8 | 1 | 12.5 |
| Citrobacter SPP | Adeyemo et al. | 2021 | Nigeria | carbapenem | 19 | 0 | 0.0 |
| Acinetobacter | Adeyemo et al. | 2021 | Nigeria | carbapenem | 9 | 4 | 44.4 |
|  | Berhanu et al. | 2021 | Ethiopia | Meropenem | 20 | 12 | 60.0 |
| Providencia Spp. | Adeyemo et al. | 2021 | Nigeria | carbapenem | 3 | 0 | 0.0 |
| Providencia rettgeri | Berhanu et al. | 2021 | Ethiopia | Meropenem | 5 | 0 | 0.0 |
| Pseudomans spp. | Hamid et al. | 2020 | Sudan | Meropenem | 3 | 2 | 66.7 |
|  | Hamid et al. | 2020 | Sudan | Imipenem | 16 | 1 | 6.3 |
| Proteus Vulgaris | Berhanu et al. | 2021 | Ethiopia | Meropenem | 15 | 0 | 0.0 |
| Enterobacter cloacae | Berhanu et al. | 2021 | Ethiopia | Meropenem | 6 | 0 | 0.0 |
| Salmonella enterica subsp. Arizonae | Adeyemo et al. | 2021 | Nigeria | carbapenem | 2 | 0 | 0.0 |
| Hafnia alvei | Adeyemo et al. | 2021 | Nigeria | carbapenem | 5 | 2 | 40.0 |
| Stenotrophomonas maltophilia | Adeyemo et al. | 2021 | Nigeria | carbapenem | 1 | 0 | 0.0 |
